# Women's involvement in decision-making and receiving husbands’ support for their reproductive healthcare: a cross-sectional study in Lalitpur, Nepal

**DOI:** 10.1093/inthealth/ihac034

**Published:** 2022-05-24

**Authors:** Alpha Pokharel, Samidha Dhungel Pokharel

**Affiliations:** Department of Women's Study, Tribhuvan University, Kathmandu, Nepal; Padma Kanya Multiple Campus, Tribhuvan University, Kathmandu, Nepal

**Keywords:** decision making, husband involvement, husbands’ support, Nepal, reproductive health services, women

## Abstract

**Background:**

Sociocultural factors remain an important determinant for women's involvement with decision making and getting husbands’ support for their reproductive healthcare. Therefore this study was conducted to examine sociodemographic factors associated with women's involvement in decision making and getting husbands’ support for their reproductive healthcare.

**Methods:**

An institutional-based cross-sectional study was conducted in Lalitpur, Nepal. A total of 600 respondents were selected from 15 immunization clinics. Participants were women ≥18 y of age who came to the child's immunization clinic. The association between sociodemographic variables and women's involvement in decision making and getting husbands’ support for their reproductive healthcare was analysed through multivariate logistic regression models.

**Results:**

While women's involvement in decision making was greater for childcare, it was less in the area related to financial matters. In contrast, husbands supported more in the area related to finances than for childcare and accompanying to health facilities. The significant determinants for women's involvement in decision making and getting husbands’ support were the woman's caste, education level, employment status, household income, age group and number of children. Madhesi/Muslim/other women were less likely (adjusted odds ratio [AOR] 0.31 [95% confidence interval {CI} 0.12 to 0.73]) to decide the number of babies and birth spacing. These women were also less likely (AOR 0.18 [95% CI 0.02 to 0.86]) to be accompanied by their husbands to the family planning (FP) clinic. Janajati, Dalit and Madhesi/Muslim/other women were less likely to receive their husbands’ support for birth preparedness. Women who were <20 y of age and had a single child were less likely to get involved in decision making and getting their husbands’ support for FP services.

**Conclusions:**

The findings call for reproductive health programs that encourage women's involvement in decision making and receiving husbands’ support in women's reproductive healthcare. When designing such a program in the FP area, the woman's caste, age and parity should be given special consideration. Also, caste should be considered when designing such a program related to birth preparedness.

## Introduction

Reproductive healthcare access and utilization are affected by many sociocultural factors, including women's involvement in decision making and getting husbands’ support for their reproductive healthcare.^1^^,^^2^ Women's involvement in decision making was found to reduce the unmet need for family planning (FP),^3^ improve antenatal care (ANC) visits,^2^ and improve birth preparedness.^4^ Despite the significance, globally one-quarter of women cannot decide on their reproductive healthcare.^5^ This is because husbands remain the sole decision maker for their wives’ reproductive healthcare access and utilization.^6^ As a result, many women are hindered from accessing and utilizing reproductive healthcare services.^6^ Also, women do not hold financial authority, are shy to be exposed to outsiders and are not educated enough to understand the instructions at the health facility.^6^ They have to rely on their husbands’ support to access and utilize reproductive healthcare services.^6^ A meta-analysis showed that the husbands’ involvement improved overall utilization of maternal health services.^1^ Also, the husbands’ support in household chores during the postnatal period improved maternal health outcomes.^7^ Therefore women's involvement in decision making and getting husbands’ support are essential to increase the utilization of reproductive health services and improve maternal health outcomes.^1^^,^^8^

Knowing the situation of women's involvement as decision makers and husbands’ involvement as supportive partners for women's reproductive healthcare are pivotal for achieving universal access to reproductive health.^1^^,^^9^ Past studies have assessed women's and their husbands’ involvement in reproductive healthcare. However, most of these studies have focused on either contraception, ANC or institutional delivery.^1^^,^^10^ Only a few studies have assessed all these components together in a single study and included childcare components such as immunization and breastfeeding.^11^^,^^12^ Further, despite having a significant impact on maternal health outcomes,^7^ little evidence exists on the situation of husbands’ support with domestic chores during pregnancy and postnatal periods. Furthermore, variations were found across husbands’ involvement in different components of reproductive health. For instance, husbands supported more in the financial area than accompanying to health facilities.^11^ As variations exist across different components of reproductive healthcare, it is crucial to analyse the components together in a single study to visualize the existing situation better.

In many countries, including Nepal, patriarchal traditions exist in many societies and sociocultural factors are important determinants for different life courses.^13^^,^^14^ The existing gender norms and sociocultural factors influence women's involvement in decision making and receiving husbands’ support for their reproductive healthcare.^14^ Knowing the sociodemographic determinants for women's involvement with decision making and getting husbands’ support will help in designing a focused and targeted program, particularly in the context of Nepal. Despite the importance, little is known about the influence of this sociodemographic characteristic on women's involvement in decision making and receiving husbands’ support over different components of reproductive health. Further, very limited evidence exists for sociodemographic determinants of husbands’ involvement in childcare and domestic chores. Considering all these research gaps and needs, this study was conducted to identify sociodemographic factors associated with women's involvement in decision making and getting husbands’ support for their reproductive healthcare (FP, ANC, birth preparedness and childcare).

## Methods

### Study design and setting

An institutional-based, cross-sectional study was conducted in the Lalitpur district of Nepal. The population of Lalitpur district is 468 132.^15^ It comprises one metropolitan city, two urban municipalities and three rural municipalities.^16^ Despite being close to the country's capital, the reproductive health indicators of Lalitpur district are not satisfactory.^17^ As the majority of the children in Nepal are taken to the immunization clinic by their mother, the immunization clinic was considered a study setting for this study.^12^^,^^18^ There are 42 government-run immunization clinics in Lalitpur district.^16^ These are static clinics located within the government-run health facility and are run by the health facility staffs. Some of these clinics also run outreach service in the community.^19^ However, for this study only the immunization clinic located at the health facility was included. These clinics run monthly, bimonthly, trimonthly or weekly, depending upon the client flow.

### Study participants and sampling

All women >18 y of age who took their child to the immunization clinic were included in the study. The national immunization program of Nepal provides routine vaccination to children from 0 to 15 months of age. Samples were selected in two stages. In the first stage, 15 of 42 government-run immunization clinics were selected through a simple random sampling method. Three immunization clinics were selected from one metropolitan city, three from each of the two urban municipalities and two from each of the two rural municipalities, making a total 15. The number of health facilities from each municipality was purposively decided for better representation. In the second stage, 40 eligible women were consecutively selected from each immunization clinic. Because immunization clinics in Nepal have a walk-in system, random sampling was not possible in the second stage. The sample size was determined using the following assumption: proportion of husbands’ involvement was 39.3%,^12^ 5% margin of error, 10% non-response rate and design effect of 1.5.^20^ The final sample size was calculated to be 597.

### Variables

#### Sociodemographic variables

The sociodemographic variables used in this study were the women's age, education level, employment status, aggregated annual household income in Nepali rupees (NPR), caste, years of marriage and the number of children.^11–13^ The categorization of the sociodemographic variables was adopted using the Nepal Demographic Health Survey (NDHS) 2016.^21^ The nominal median household income of 127 281 NPR from the Nepal Living Standard Survey 2010/11 was considered the cut-off point for the aggregated annual household income.^22^ The median value was considered a cut-off point for the duration of the marriage and the number of children.

#### Outcome variables

The main outcome variables were women's involvement in decision making (yes/no) and receiving husbands’ support for their reproductive healthcare (yes/no).

#### Women's involvement in decision making

A woman was considered to be involved in decision making for her reproductive healthcare if decision making was done alone or jointly with her husband. The women's involvement in decision making was assessed for contraceptive method, choice of FP clinic, the number of babies and birth spacing, having an antenatal check-up, choice of the birthing centre, choice of transportation to the birthing centre, having exclusive breastfeeding and immunization of the child.

#### Receiving husbands’ support for women's reproductive healthcare

A woman was considered to receive her husband's support if she responded ‘yes’ to the questions related to it. Receiving husbands’ support was assessed for accompanying to the FP clinic, accompanying to the antenatal clinic, helping with domestic chores during pregnancy, saving money for childbirth expenses, arranging the birthing centre, arranging transportation to the birthing centre, assisting with breastfeeding, helping with domestic chores during the postnatal period and accompanying to the child immunization clinic. Women whose husbands were not present (outmigrants) during the period of pregnancy, childbirth and child care were excluded from the analysis. However, exclusion was not applied for saving money, as it was possible without physical presence.

### Validity and reliability

The questionnaire items were adapted from survey tools and indicators for maternal and newborn health developed by Jhpiego, an affiliate of Johns Hopkins University,^23^ NDHS^21^ and relevant prior studies.^11–13^ The questionnaire consisted of sociodemographic characteristics of the women, women's involvement in decision making and receiving husbands’ support for their reproductive healthcare. As the questionnaires were not available in the Nepali language, they were checked for cultural adaptation through translation and back-translation to the Nepali language and pretesting of the questionnaire. The content validity was ensured through two expert opinions, two focus group discussions (FGDs) among women and pretest of the questionnaires. The pretest of the questionnaire was done among 60 respondents in the Chhetrapati Family Welfare Clinic, Kathmandu. The value of Cronbach's α was 0.84 for total decision-making questionnaires (eight items) and 0.91 for receiving husbands’ support (nine items). Necessary modifications were made from the result of the pretest and FGDs.

### Data collection

The tool for data collection was a structured questionnaire. The data collection was done from November 2018 to February 2019. Three research assistants were hired for data collection. The research assistants were trained and supervised for data collection.

### Data analysis

Descriptive statistics were used to summarize the sociodemographic characteristics of women, women's involvement in decision making and receiving husbands’ support for their reproductive healthcare. Multicollinearity was checked through the variance inflation factor. None of the sociodemographic variables were found to be multicollinear. Multivariate logistic regression analysis was used to identify the association of sociodemographic variables with women's involvement in decision making and receiving husbands’ support for their reproductive healthcare. Eight different models for decision making and nine different models for receiving husbands’ support were run. The models included the variables with theoretical or rational associations from prior studies.^11^^,^^12^ The significance level was set at 0.05 and analysis was conducted using R Studio version 1.2.5001 (R Studio, Boston, MA, USA).

## Results

### Background characteristics of the women

Table 1 shows the background characteristics of the women. A total of 600 women participated in the survey. A total of 514 (85.7%) women were >20 y of age. There was an equal percentage distribution of women for different categories of education level. Of 600 women, 25.3% of the women had no education and 26.1% had an education level above a school leaving certificate (SLC). More than two-thirds of the women (79.0%) worked as non-wage earners and 85.0% had an aggregated annual income >127 281 NPR. About half of the women belonged to the Janajati caste (49.0%). More than half of the women were married for <2 y (60.3%) and had a single child (53.0%).

**Table 1.** Background characteristics of women (N=600)

Characteristics	n (%)
Age (years)	
<20	86 (14.3)
>20	514 (85.7)
Educational level	
No education	152 (25.3)
Primary	148 (24.6)
Secondary	143 (23.8)
SLC and above	157 (26.1)
Employment status	
Wage earner	126 (21.0)
Non-wage earner	474 (79.0)
Aggregated annual household income (NPR)^a^	
<127 281	90 (15.0)
>127 281	510 (85.0)
Caste	
Brahmin and Chettri^b^	200 (33.3)
Janajati^c^	294 (49.0)
Dalit^d^	78 (13.0)
Madhesi/Muslim/other^e^	28 (4.6)
Years of marriage	
0–2	362 (60.3)
>2	238 (39.6)
Number of children	
0–1	318 (53.0)
>1	282 (47.0)

a US$1=119.89 NPR.

^b^Considered to be of a higher caste in Nepali society.

^c^Indigenous group.

^d^Considered to be the lowest caste in Nepali society.

^e^Group of marginalized and minority caste in Nepal.

### Women's involvement in decision making for their reproductive healthcare

Table 2 presents women's involvement in decision making for their reproductive healthcare. About half of the women (51.3%) were involved in decision making for contraceptive method. Of the 600 women, 39.6% were involved in deciding the choice of a FP clinic and 59.1% were involved in decision making for the number of children and birth spacing. A total of 316 (52.6%) were involved in decision making to have an ANC check-up. Half of the women (51.8%) were not involved in decision making for the choice of the birthing centre and 74.3% were not involved in the choice of transportation to the birthing centre. About two-thirds (67.0%) were involved in decision making for having exclusive breastfeeding and 81.0% of them were involved in the decision to immunize the child.

**Table 2.** Women's involvement in decision making regarding their reproductive healthcare (N=600)

	FP			ANC and birth preparedness			Childcare	
Decision making	Choice of contraception, n (%)	Choice of FP clinic, n (%)	Number of children and birth spacing, n (%)	Having an ANC check-up, n (%)	Choice of birthing centre, n (%)	Choice of transportation to birthing centre, n (%)	Exclusive breastfeeding, n (%)	Immunization of the child, n (%)
Women involved	308 (51.3)	238 (39.6)	355 (59.1)	316 (52.6)	289 (48.1)	154 (25.6)	402 (67.0)	486 (81.0)
Women not involved	192 (32.0)	226 (37.6)	233 (38.8)	284 (47.3)	311 (51.8)	446 (74.3)	198 (33.0)	114 (19.0)
Not decided yet	100 (16.6)	136 (22.6)	12 (2.0)	NA	NA	NA	NA	NA

NA: not available.

### Receiving husbands’ support for women's reproductive healthcare

Table 3 presents the husbands’ support for women's reproductive healthcare. Among 600 women, 337 had used or were using a female modern contraceptive method. Among the 337 women, 69.4% were never accompanied by their husbands to the FP clinic. Of 600 women, 56.0% were accompanied by their husbands to the ANC clinic and 59.0% were helped with domestic chores during pregnancy. The majority of the women (84.5%) were supported in saving money for childbirth expenses by their husbands. About two-thirds of the women's husbands helped arrange the birthing centre (65.3%) and transportation to the birthing centre (64.6%). About half of the women did not receive their husbands’ support for breastfeeding (48.8%) and were not accompanied to the immunization clinic (57.5%). More than half (58.1%) of the women were supported by their husbands for domestic chores during the postnatal period. Only 8.0% of the women said their husbands were not present during the pregnancy and childcare period.

**Table 3.** Husbands’ support for women's reproductive healthcare

	FP (n=337)	ANC and birth preparedness (n=600)					Childcare (n=600)		
Received husbands’ support	Accompanied to FP clinic, n (%)	Accompanied to ANC clinic, n (%)	Helped with domestic chores, n (%)	Saved money for childbirth expenses, n (%)	Arranged the birthing centre, n (%)	Arranged transportation to the birthing centre, n (%)	Supported with breastfeeding, n (%)	Accompanied to immunization clinic, n (%)	Helped with domestic chores, n (%)
Yes	103 (30.5)	336 (56.0)	354 (59.0)	507 (84.5)	392 (65.3)	388 (64.6)	259 (43.1)	207 (34.5)	349 (58.1)
No	234 (69.4)	217 (36.1)	199 (33.1)	93 (15.5)	160 (26.6)	164 (27.3)	293 (48.8)	345 (57.5)	203 (33.8)
Not present	NA	47(7.8)	47 (7.8)	NA	48 (8.0)	48 (8.0)	48 (8.0)	48 (8.0)	48 (8.0)

### Sociodemographic factors associated with women's involvement in decision making for their reproductive healthcare

Table 4 shows the association of sociodemographic factors with women's involvement in decision making for their reproductive healthcare by multivariate logistic regression analysis. There are a total of eight models for each question of decision making.

Women >20 y of age were more likely to be involved in decision making for choice of transportation (AOR 2.33 [95% CI 1.13 to 5.22]) and having exclusive breastfeeding (AOR 1.95 [95% CI 1.02 to 3.57]) than women <20 y of age. Women with an aggregated annual household income >127 281 NPR were more likely to have an ANC check-up (AOR 2.19 [95% CI 1.31 to 3.72]) than women whose aggregated annual household income was <127 281 NPR. Women with an education level above an SLC were more likely to be involved in all eight decision-making components than women with no education. Women who were wage earners were more likely to decide on the choice of birthing centre (AOR 1.85 [95% CI 1.21 to 2.87]) and the choice of transportation to the birthing centre (AOR 1.66 [95% CI 1.05 to 2.60]) than non-wage earners.

**Table 4.** Sociodemographic factors associated with women's involvement in decision making for their reproductive healthcare

	FP, AOR (95% CI)			ANC and birth preparedness, AOR (95% CI)			Childcare, AOR (95% CI)	
Sociodemographic variables	Choice of contraception (n=500)	Choice of FP clinic (n=464)	Number of children and birth spacing (n=588)	Having an ANC check-up (n=600)	Choice of birthing centre (n=600)	Choice of transportation (n=600)	Exclusive breastfeeding (n=600)	Immunization of the child (n=600)
Women's age (years)								
<20	ref	ref	ref	ref	ref	ref	ref	ref
>20	0.72 (0.39 to 1.32)	0.79 (0.43 to 1.45)	0.64 (0.36 to 1.10)	1.20 (0.70 to 2.09)	0.69 (0.40 to 1.20)	2.33* (1.13 to 5.22)	1.95* (1.02 to 3.57)	1.00 (0.57 to 1.74)
Annual household income (NPR)^a^								
<127 281	ref	ref	ref	ref	ref	ref	ref	ref
>127 281	0.88 (0.48 to 1.57)	0.97 (0.54 to 1.75)	1.65 (0.98 to 2.80)	2.19** (1.31 to 3.72	1.28 (0.76 to 2.18)	1.24 (0.68 to 2.37)	1.02 (0.74 to 1.46)	1.28 (0.76 to 2.12)
Women's education level								
No education	ref	ref	ref	ref	ref	ref	ref	ref
Primary	2.91*** (1.66 to 5.19)	1.60 (0.90 to 2.85)	1.12 (0.67 to 1.86)	0.68 (0.41 to 1.14)	1.35 (0.80 to 2.27)	1.13 (0.60 to 2.13)	0.97* (1.54 to 1.98)	2.18** (1.31 to 3.64)
Secondary	5.25*** (2.90 to 9.70)	3.14*** (1.68 to 5.96)	2.51*** (1.47 to 4.33)	2.13** (1.28 to 3.59)	1.82* (1.09 to 3.10)	0.92 (0.48 to 1.73)	1.26 (0.70 to 2.30)	3.04*** (1.79 to 5.23)
SLC and above	8.24*** (4.28 to 15.42)	4.16*** (2.28 to 7.73)	3.72*** (2.10 to 6.72)	3.07*** (1.76 to 5.41)	3.45*** (1.97 to 6.11)	2.65** (1.43 to 4.97)	2.04* (1.15 to 4.64)	5.34*** (2.93 to 9.96)
Women's employment status								
Non-wage earner	ref	ref	ref	ref	ref	ref	ref	ref
Wage earner	0.95 (0.58 to 1.56)	1.41 (0.87 to 2.28)	1.07 (0.69 to 1.69)	1.15 (0.75 to 1.77)	1.85** (1.21 to 2.87)	1.66* (1.05 to 2.60)	1.00 (0.59 to 1.75)	0.87 (0.55 to 1.37)
Caste								
Brahmin/Chettri^b^	ref	ref	ref	ref	ref	ref	ref	ref
Janajati^c^	0.72 (0.45 to 1.15)	0.56 (0.35 to 0.88)	0.56** (0.36 to 0.86)	1.09 (0.72 to 1.64)	0.61* (0.40 to 0.90)	0.82 (0.52 to 1.28)	0.60 (0.34 to 1.02)	0.78 (0.58 to 1.42)
Dalit^d^	0.70 (0.36 to 1.38)	0.79 (0.41 to 1.52)	0.67 (0.36 to 1.25)	1.01 (0.55 to 1.84)	0.53* (0.29 to 0.95)	1.08 (0.54 to 2.13)	0.60 (0.28 to 1.26)	0.92 (0.50 to 1.21)
Madhesi/Muslim/other^e^	1.96 (0.73 to 3.95)	0.79 (0.30 to 2.11)	0.31** (0.12 to 0.73)	0.48 (0.19 to 1.15)	0.20** (0.07 to 0.51)	0.72 (0.24 to 1.87)	2.38 (0.61 to 15.87)	1.63 (0.62 to 4.87)
Years of marriage								
0–2	1.04 (0.55 to 2.00)	1.21 (0.65 to 2.28)	0.71 (0.40 to 1.23)	0.85 (0.50 to 1.44)	0.62 (0.36 to 1.05)	0.64 (0.43 to 1.08)	0.67 (0.45 to 1.15)	0.69 (0.39 to 1.22)
>2	ref	ref	ref	ref	ref	ref	ref	ref
Number of children								
0–1	0.41* (0.21 to 0.78)	0.42** (0.22 to 0.78)	0.61 (0.35 to 1.05)	0.93 (0.55 to 1.56)	1.00 (0.59 to 1.71)	1.06 (0.69 to 1.79)	1.02 (0.58 to 1.76)	0.98 (0.56 to 1.69)
>1	ref	ref	ref	ref	ref	ref	ref	ref

ref: reference.

a US$1=119.89 NPR.

^b^Considered to be of a higher caste in Nepali society.

^c^Indigenous group.

^d^Considered to be the lowest caste in Nepali society.

^e^Group of marginalized and minority caste of Nepal.

*p<0.05, **p<0.01, ***p<0.001.

Women who belonged to the Janajati (AOR 0.56 [95% CI 0.36 to 1.25]) and Madhesi/Muslim/other (AOR 0.31 [95% CI 0.12 to 0.73]) were less likely to decide on the number of babies and birth spacing than those of the Brahmin and Chettri castes. Similarly, the Janajati (AOR 0.61 [95% CI 0.40 to 0.90]), Dalit (AOR 0.53 [95% CI 0.29 to 0.95]) and Madhesi/Muslim/other (AOR 0.20 [95% CI 0.07 to 0.51]) were also less likely to decide on the choice of birthing centre than women who belonged to the Brahmin and Chettri castes. Women having a single child were less likely to decide on the choice of contraceptive method (AOR 0.41 [95% CI 0.21 to 0.78]) and the FP clinic (AOR 0.42 [95% CI 0.22 to 0.78]) than women who have more than one child.

### Sociodemographic factors associated with receiving husbands’ support for women's reproductive healthcare

Table 5 presents the association of sociodemographic factors with receiving their husbands’ support for women's reproductive healthcare by multivariate logistic regression analysis. There are nine models for each question of receiving their husbands’ support. Women <20 y of age were less likely (AOR 0.38 [95% CI 1.14 to 0.95]) to be accompanied by their husband for the FP clinic than women >20 y of age. Women with an aggregated annual household income >127 281 NPR were more likely to be accompanied to the FP clinic (AOR 2.31 [95% CI 1.43 to 4.59]) and have money saved (AOR 3.50 [95% CI 1.95 to 6.23]) by their husbands than women with an aggregated annual household income <127 281 NPR. Women with an education level above an SLC were more likely to receive support from their husbands in all nine components than women with no education.

**Table 5.** Sociodemographic factors associated with receiving husbands’ support for women's reproductive healthcare

	FP (n=337), AOR (95% CI)	ANC and birth preparedness (n=600), AOR (95% CI)					Childcare (n=600), AOR (95% CI)		
Sociodemographic variables	Accompanied to FP clinic	Accompanied to ANC clinic	Helped with domestic chores	Saved money	Arranged the birthing centre	Arranged transportation	Supported with breastfeeding	Accompanied to immunization clinic	Helped with domestic chores
Women's age (years)									
<20	ref	ref	ref	ref	ref	ref	ref	ref	ref
>20	0.38* (0.14 to 0.95)	0.76 (0.41 to 1.37)	0.90 (0.49 to 1.61)	1.33 (0.65 to 2.65)	1.08 (0.41 to 2.63)	1.38 (0.75 to 2.51)	0.96 (0.56 to 1.63)	1.09 (0.62 to 1.94)	1.16 (0.65 to 2.06)
Aggregated annual household income (NPR)^a^									
<127 281	ref	ref	ref	ref	ref	ref	ref	ref	ref
>127 281	2.31** (1.43 to 4.59)	1.14 (0.66 to 1.97)	1.24 (0.56 to 1.63)	3.50* (1.95 to 6.23)	1.67 (0.96 to 2.86)	1.66 (0.96 to 2.85)	0.92 (0.55 to 1.53)	1.25 (0.70 to 2.27)	1.51 (0.88 to 2.56)
Women's education level									
No education	ref	ref	ref	ref	ref	ref	ref	ref	ref
Primary	0.79 (0.33 to 1.86)	2.84*** (1.68 to 4.87)	2.04** (1.21 to 3.45)	0.96 (0.50 to 1.83)	1.39 (0.81 to 2.39)	1.09 (0.64 to 1.86)	1.07 (0.63 to 1.80)	1.22 (0.67 to 2.24)	1.82* (1.08 to 3.08)
Some secondary	3.63** (1.65 to 8.35)	5.63*** (3.11 to 10.41)	4.07*** (2.32 to 7.26)	1.17 (0.59 to 2.38)	2.56*** (1.37 to 4.85)	2.35** (1.26 to 4.44)	2.14** (1.28 to 3.62)	2.97*** (1.65 to 5.41)	3.96*** (2.23 to 7.19)
SLC and above	5.42*** (2.26 to 13.58)	8.15*** (4.53 to 15.08)	5.90*** (3.19 to 11.18)	1.90 (0.85 to 4.46)	3.16* (1.73 to 5.91)	2.99*** (1.64 to 5.57)	2.68*** (1.56 to 4.67)	4.33*** (2.36 to 8.11)	4.12*** (2.28 to 7.57)
Women's employment status									
Non-wage earner	ref	ref	ref	ref	ref	ref	ref	ref	ref
Wage earner	1.78 (0.91 to 3.48)	0.98 (0.62 to 1.57)	1.30 (0.81 to 2.12)	0.99 (0.55 to 1.84)	1.28 (0.79 to 2.13)	1.02 (1.63 to 1.66)	1.03 (0.67 to 1.57)	1.40 (0.86 to 2.27)	1.78 (0.98 to 2.97)
Caste									
Brahmin/Chettri^b^	ref	ref	ref	ref	ref	ref	ref	ref	ref
Janajati^c^	0.84 (0.45 to 1.57)	0.67 (0.42 to 1.05)	0.84 (0.53 to 1.34)	0.47* (0.24 to 0.89)	0.47** (0.40 to 0.90)	0.50* (0.30 to 0.81)	1.01 (0.68 to 1.50)	1.03 (0.66 to 1.60)	0.88 (0.55 to 1.39)
Dalit^d^	0.31* (0.10 to 0.84)	1.25 (0.65 to 2.43)	0.73 (0.38 to 1.40)	0.27** (0.12 to 0.60)	0.66* (0.29 to 0.95)	0.77* (0.39 to 0.98)	0.92 (0.50 to 1.65)	0.79 (0.41 to 1.52)	0.69 (0.37 to 1.29)
Madhesi/Muslim/other^e^	0.18* (0.02 to 0.86)	1.12 (0.45 to 2.93)	0.53 (0.20 to 1.36)	0.29* (0.09 to 0.92)	0.28** (0.07 to 0.51)	0.31* (0.12 to 0.79)	0.77 (0.31 to 1.83)	0.31 (0.08 to 1.01)	0.54 (0.22 to 1.36)
Years of marriage									
0–2	1.89 (0.78 to 4.67)	1.22 (0.69 to 2.16)	1.64 (0.91 to 2.98)	0.81 (0.39 to 1.71)	1.59 (0.36 to 1.95)	2.18* (1.19 to 4.08)	1.59 (0.96 to 2.66)	1.77 (0.98 to 3.18)	1.49 (0.83 to 2.70)
>2	ref	ref	ref	ref	ref	ref	ref	ref	ref
Number of children									
0–1	0.36* (0.14 to 0.91)	0.84 (0.47 to 1.46)	0.56 (0.31 to 1.01)	1.33 (0.63 to 2.72)	0.58 (0.59 to 1.71)	0.71 (0.38 to 1.31)	0.95 (0.58 to 1.56)	1.22 (0.69 to 2.16)	0.84 (0.46 to 1.50)
>1	ref	ref	ref	ref	ref	ref	ref	ref	ref

ref: reference.

a US$1=119.89 NPR.

^b^Considered to be of a higher caste in Nepali society.

^c^Indigenous group.

^d^Considered to be the lowest caste in Nepali society.

^e^Group of marginalized and minority caste of Nepal.

*p<0.05, **p<0.01, ***p<0.001.

Women who belonged to the Dalit (AOR 0.31 [95% CI 0.10 to 0.84]) and Madhesi/Muslim/other (AOR 0.18 [95% CI 0.02 to 0.86]) were less likely to be accompanied by their husbands to the FP clinic. Women who belonged to the Janajati, Dalit and Madhesi/Muslim/other were less likely to receive their husbands’ support in saving money for the birth expenses, arranging the birthing centre and arranging transportation to the birthing centre than those of the Brahmin and Chettri castes. Women married for <2 y were more likely (AOR 2.18 [95% CI 1.19 to 4.08]) to receive their husbands’ support for arranging transportation to the birthing centre than women married >2 y. Women having a single child were less likely (AOR 0.36 [95% CI 0.14 to 0.91]) to be accompanied by their husbands to the FP clinic than women with more than one child.

## Discussion

Women's involvement in decision making was more in the area related to childcare than finances. In contrast, husbands supported more in the area pertaining to finances than for childcare and accompanying to health facilities. 

This study showed that women were less involved in decision making that included financial matters, such as having an ANC check-up, birthing centre choice and choice of transportation to the birthing centre. In contrast, their involvement was higher for childcare, such as deciding on immunization and breastfeeding. The possible explanation could be due to the existing gender norms of society. The financial burden of healthcare is shouldered by men, but childcare is exclusively considered a women's responsibility.^14^ Past studies found a significant association of women involved in decision making and increased maternal health service utilization.^3^^,^^8^^,^^24^ Therefore, women's involvement in decision making for their reproductive healthcare should be encouraged, focusing on components related to financial matters, such as the choice of transportation and birthing centre.

Less support was received from husbands for accompanying to the FP clinic, ANC clinic and child's immunization clinic. Similar findings were observed in past studies.^11^^,^^25^ Some of the possible barriers could be male-unfriendly reproductive health facilities, the negative attitude of healthcare providers and the perception that it is exclusively a woman's activity.^26^^,^^27^ This study also found that more women received support from their husbands for reproductive healthcare related to financial matters than childcare. This could be because of the existing gender norms of society, where childcare is considered a woman's responsibility.^14^ Also, the role of mother-in-law as a primary caretaker during the postnatal period could have made it unnecessary for husbands to be involved in childcare.^14^ However, a past study showed that women want their husbands to get involved in childcare.^28^ Also, a prior systematic review found a significant association between husbands’ support and women's reproductive health service utilization.^1^ Therefore, programs that encourage husbands to accompany their wives to the reproductive health and immunization clinics should be considered, as well as programs that encourage their participation in childcare activities.

This study showed that Brahmin and Chettri women were more likely to get involved in decision making regarding the number of children and birth spacing and were accompanied by their husbands to FP compared with the other castes. The 2016 NDHS shows no significant differences among the castes for the unmet need for FP.^21^ However, another study from Nepal shows a high unmet need among Muslims.^29^ The Dalit, Janajati and Madhesi/Muslims/other are considered lower castes than the Brahmin and Chettri by traditional Nepali society.^30^ These caste groups have lower literacy status and higher poverty levels than the Brahmin and Chettri.^30^ In addition to lower literacy and income level, not being involved in decision making and getting husbands’ support for FP service uptake may further decrease their rate of FP service utilization. Thus further consideration should be given to the caste when designing programs related to increasing women's involvement in decision making and getting husbands’ support for FP service.

It was also evidenced from this study that women belonging to the Dalit, Janajati and Madhesi/Muslim/other were less likely to get involved in decision making in choosing the birthing centre. Also, these groups of women were less likely to get support from husbands for birth preparedness (saving money, arranging the birthing centre, arranging transportation to the birthing centre). The national data of Nepal reports lower reproductive health service utilization among these groups of women compared with the Brahmin and Chettri.^21^ One important strategy to increase reproductive health services utilization and prevent obstetric emergencies is to have a birth preparedness plan.^11^ Not being involved in decision making and getting husbands’ support for birth preparedness may further decrease their reproductive health service utilization rate and increase obstetric emergencies. Therefore, when designing a program to improve husbands’ involvement in women's reproductive healthcare, special consideration should be given to the caste.

Women <20 y of age and who had a single child were less likely to be accompanied by their husband to the FP clinic. Similarly, women who had a single child were less likely to get involved in decision making in the choice of contraception and the FP clinic. The 2016 NDHS data reports a higher unmet need among women who were young and had fewer children.^21^ A prior study found a lack of women's involvement in decision making and husbands’ involvement as a key determinant for high unmet need for FP.^31^ Therefore, when designing an FP program to encourage women's decision making and husbands’ involvement in FP service utilization, special consideration should be given to young women.

This study also found that women with a higher annual household income were more likely to get involved in decision making for ANC check-ups. These women were also more likely to receive their husbands’ support for accompanying to the FP clinic and saving money. The decision to have an ANC check-up and transportation certainly involves financial matters, and having a higher income would undoubtedly facilitate these decisions.^32^ Further, receiving husbands’ support to save money and being accompanied to an FP clinic is possible when income is higher.^12^ Therefore, further consideration should be given to women with lower household incomes when designing such a program.

Women who were wage earners were more likely to get involved in decision making for the choice of the birthing centre and transportation to the birthing centre. A similar result was found in past studies in Nepal and other countries.^13^^,^^32^ Working women have greater financial independence and do not depend upon their husbands for decision making.^32^ Having financial independence enables women to choose the birthing centre and transportation to it.^13^ Also, in this study, no significant association was found for being a wage earner and greater support from husbands. However, a past study found it to be significant.^32^ Studies that explore the underlying reason are recommended.

Women who had higher education were more likely to be involved in decision making and receiving husbands’ support for their reproductive healthcare. Similar findings were present in past studies.^12^^,^^32^ Education empowers women to make decisions about their reproductive health and receive support from their husbands.^32^

### Limitations

This study might have selection bias. Women coming to child immunization clinics are more likely to utilize reproductive healthcare services, thus this study might not have captured the women who did not utilize the child immunization services, were sick or whose family members took the responsibility of bringing the child to the immunization clinic. However, since the immunization coverage is high and the majority of the children in Nepal are brought to immunization clinics by their mothers, the selection bias is likely to be limited.^18^^,^^21^ There may also be social desirability bias while reporting the answers for decision making and receiving husbands’ support. We tried to overcome this by taking data from women alone in a secure place. Lastly, whether women want their husband to be involved as a decision maker and supporter was not assessed in this study. Also, the underlying reason behind decreased involvement in decision making and receiving their husbands’ support was not explored.

### Conclusions

While women's involvement in decision making was higher for childcare, it was less in the areas related to financial matters. More husbands’ support was received in the areas related to finances than for childcare and accompanying to health facilities. These findings call for programs that encourage women's involvement in decision making and husbands’ support for women's reproductive healthcare. Also, significant determinants for women's involvement in decision making and receiving husbands’ support were caste, education level, employment status, household income, age group and the number of children. These sociodemographic characteristics should be considered when designing programs to strengthen women's decision making involvement and getting husbands’ support for their reproductive healthcare. When designing such a program in the FP area, emphasis should be given to caste, age of the women and parity. Also, caste should be particularly considered when designing programs related to birth preparedness.

## Data Availability

The data that support the findings of this study are available from the corresponding author upon reasonable request.

## References

[1] Yargawa J , Leonardi-Bee J. Male involvement and maternal health outcomes: systematic review and meta-analysis. J Epidemiol Community Health. 2015;69(6):604–12. 25700533 10.1136/jech-2014-204784 PMC4453485

[2] Zhang Z , Cunningham K, Adhikari RP et al. Maternal decision-making input and health-seeking behaviors between pregnancy and the child's second birthday: a cross-sectional study in Nepal. Matern Child Health J. 2020;24(9):1121–9. 32557134 10.1007/s10995-020-02961-z PMC7419350

[3] Sougou NM , Bassoum O, Faye A et al. Women's autonomy in health decision-making and its effect on access to family planning services in Senegal in 2017: a propensity score analysis. BMC Public Health. 2020;20:872. 32503492 10.1186/s12889-020-09003-x PMC7275346

[4] Kabakyenga JK , Östergren PO, Turyakira E et al. Influence of birth preparedness, decision-making on location of birth and assistance by skilled birth attendants among women in south-western Uganda. PLoS One. 2012;7(4):e35747. 22558214 10.1371/journal.pone.0035747 PMC3338788

[5] United Nations Population Fund . UNFPA study shows limits on women's reproductive decision-making worldwide – one quarter of women cannot refuse sex. Available from: https://www.unfpa.org/press/unfpa-study-shows-limits-womens-reproductive-decision-making-worldwide-one-quarter-women[accessed 21 March 2022].

[6] Dudgeon MR , Inhorn MC. Men's influences on women's reproductive health: medical anthropological perspectives. Soc Sci Med. 2004;59(7):1379–95. 15246168 10.1016/j.socscimed.2003.11.035

[7] Bhusal BR , Bhandari N. Identifying the factors associated with depressive symptoms among postpartum mothers in Kathmandu, Nepal. Int J Nurs Sci. 2018;5(3):268–74. 31406836 10.1016/j.ijnss.2018.04.011 PMC6626200

[8] Hou X , Ma N. The effect of women's decision-making power on maternal health services uptake: evidence from Pakistan. Health Policy Plan. 2013;28(2):176–84. 22522771 10.1093/heapol/czs042

[9] United Nations Population Fund . Women's ability to decide: Issue Brief on Indicator 5.6.1 of the Sustainable Development Goals. Available from: https://www.unfpa.org/resources/womens-ability-decide-issue-brief-indicator-561-sustainable-development-goals[accessed 21 March 2022].

[10] Yuill C , McCourt C, Cheyne H et al. Women's experiences of decision-making and informed choice about pregnancy and birth care: A systematic review and meta-synthesis of qualitative research. BMC Pregnancy Childbirth. 2020;20:1–21. 10.1186/s12884-020-03023-6 PMC7285707 32517734

[11] Wai KM , Shibanuma A, Oo NN et al. Are husbands involving in their spouses’ utilization of maternal care services? a cross-sectional study in Yangon, Myanmar. PLoS One. 2015;10(12):e0144135. 26641891 10.1371/journal.pone.0144135 PMC4671588

[12] Bhatta DN. Involvement of males in antenatal care, birth preparedness, exclusive breast feeding and immunizations for children in Kathmandu, Nepal. BMC Pregnancy Childbirth. 2013;13:14. 23324410 10.1186/1471-2393-13-14 PMC3558464

[13] Acharya DR , Bell JS, Simkhada P et al. Women's autonomy in household decision-making: a demographic study in Nepal. Reprod Health. 2010;7:15. 20630107 10.1186/1742-4755-7-15 PMC2914657

[14] Mullany BC. Barriers to and attitudes towards promoting husbands’ involvement in maternal health in Katmandu, Nepal. Soc Sci Med. 2006;62(11):2798–809. 16376007 10.1016/j.socscimed.2005.11.013

[15] Government of Nepal, National Planning Commission Secreteriate, Cental Bureau of Statistics . Nepal Population and Housing Census 2011 (national report). Kathmandu, Nepal: Central Bureau of Statistics; 2014.

[16] Government of Nepal, Ministry of Home Affairs, District Administrative Office Lalitpur . District introduction. Available from: https://daolalitpur.moha.gov.np/en/page/ja-l-l-para-caya-11[accessed 18 January 2021].

[17] Khakurel D. Women's sexual reproductive health (SRH) practices in southern Lalitpur. Available from: http://hdl.handle.net/123456789/615[accessed 18 January 2021].

[18] Abdulmalek L. Parental factors affecting child's immunization status in Benghazi, Libya. Libyan J Med Sci. 2017;1(2):40–2.

[19] Government of Nepal, Ministry of Health and Population, Department of Health Service . Annual Report Department of Health Service 2076/77 (2019/2020). Kathmandu: Government of Nepal, Ministry of Health and Population, Department of Health Service; 2020.

[20] Kassa M , Abajobir AA, Gedefaw M. Level of male involvement and associated factors in family planning services utilization among married men in Debremarkos town, northwest Ethiopia. BMC Int Health Hum Rights. 2014;14:33. 25439300 10.1186/s12914-014-0033-8 PMC4268790

[21] Ministry of Health, New ERA, DHS Program, ICF . 2016 Nepal Demographic and Health Survey. Kathmandu, Nepal: Ministry of Health Nepal; 2017.

[22] Central Bureau of Statistics, National Planning Commission Secretariat, Government of Nepal . Nepal living standards survey 2010/11 (statistical report, volume two). Kathmandu, Nepal: Central Bureau of Statistics; 2011.

[23] Johns Hopkins Bloomberg School of Public Health, Family Care International, Maternal and Neonatal Health Program. Monitoring birth preparedness and complication readiness tools and indicators for maternal and newborn health. Baltimore: JHPIEGO; 2004.

[24] Adhikari R. Effect of women's autonomy on maternal health service utilization in Nepal: a cross sectional study. BMC Womens Health. 2016;16:26. 27177683 10.1186/s12905-016-0305-7 PMC4867085

[25] Chekole MK , Kahsay ZH, Medhanyie AA et al. Husbands’ involvement in family planning use and its associated factors in pastoralist communities of Afar, Ethiopia. Reprod Health. 2019;16:33. 30885215 10.1186/s12978-019-0697-6 PMC6423846

[26] Adelekan A , Omoregie P, Edoni E. Male involvement in family planning: challenges and way forward. Int J Popul Res. 2014;2014:416457.

[27] Suryawanshi D , Rajaseharan D, Venugopal R. Involvement of husband in maternal and child health care in rural field practice area of a tertiary medical college in South India—a mixed method study. J Family Med Prim Care. 2021;10(8):2829–33. 34660413 10.4103/jfmpc.jfmpc_2342_20 PMC8483132

[28] Rahman AE , Perkins J, Salam SS et al. What do women want? An analysis of preferences of women, involvement of men, and decision-making in maternal and newborn health care in rural Bangladesh. BMC Pregnancy Childbirth. 2020;20:169. 32183744 10.1186/s12884-020-2854-x PMC7079480

[29] Mehata S , Paudel YR, Mehta R et al. Unmet need for family planning in Nepal during the first two years postpartum. Biomed Res Int. 2014;2014:649567. 25003125 10.1155/2014/649567 PMC4066713

[30] Department for International Development, World Bank . Unequal citizens: gender, caste and ethnic exclusion in Nepal. Kathmandu, Nepal: World Bank Nepal; 2006.

[31] Letamo G , Navaneetham K. Levels, trends and reasons for unmet need for family planning among married women in Botswana: a cross-sectional study. BMJ Open. 2015;5(3):e006603. 10.1136/bmjopen-2014-006603 PMC4386234 25829370

[32] Darteh EKM , Dickson KS, Doku DT. Women's reproductive health decision-making: a multi-country analysis of demographic and health surveys in sub-Saharan Africa. PLoS One. 2019;14(1):e0209985. 30625212 10.1371/journal.pone.0209985 PMC6326492

